# The Epigenetics of Alzheimer’s Disease: Factors and Therapeutic Implications

**DOI:** 10.3389/fgene.2018.00579

**Published:** 2018-11-30

**Authors:** Xiaolei Liu, Bin Jiao, Lu Shen

**Affiliations:** ^1^Department of Neurology, Xiangya Hospital, Central South University, Changsha, China; ^2^The Center of Gerontology and Geriatrics, West China Hospital, Sichuan University, Chengdu, China; ^3^National Clinical Research Center for Geriatric Disorders, Central South University, Changsha, China; ^4^Key Laboratory of Hunan Province in Neurodegenerative Disorders, Central South University, Changsha, China; ^5^Key Laboratory of Organ Injury, Aging and Regenerative Medicine of Hunan Province, Changsha, China

**Keywords:** Alzheimer’s disease (AD), epigenetics, non-coding RNA (ncRNA), DNA methylation, histone modifications and chromatin structure

## Abstract

Alzheimer’s disease (AD) is a well-known neurodegenerative disorder that imposes a great burden on the world. The mechanisms of AD are not yet fully understood. Current insight into the role of epigenetics in the mechanism of AD focuses on DNA methylation, remodeling of chromatin, histone modifications and non-coding RNA regulation. This review summarizes the current state of knowledge regarding the role of epigenetics in AD and the possibilities for epigenetically based therapeutics. The general conclusion is that epigenetic mechanisms play a variety of crucial roles in the development of AD, and there are a number of viable possibilities for treatments based on modulating these effects, but significant advances in knowledge and technology will be needed to move these treatments from the bench to the bedside.

## Introduction

Alzheimer’s disease (AD), an irreversible and progressive neurological disease, is the most common form of neurodegenerative dementia (Mckhann et al., 2011; Rocca et al., 2011; Lansdall et al., 2017). The most prominent early symptom is short-term memory loss. As the disease progresses, other symptoms such as personality changes, apathy and language problems emerge. 95 percent of patients who are hospitalized with AD have the sporadic form, which is late-onset Alzheimer’s disease (LOAD) (Diniz et al., 2017; Zhao et al., 2017). The pathology of LOAD is multi-factorial with biological, genetic and environmental factors interacting with each other to aggravate the process of AD. Many genetic risk factors for LOAD have been identified. Among them, the 𝜀4 isoform of apolipoprotein E (ApoE4) is well-known as the strongest genetic risk factor for LOAD (Lane-Donovan and Herz, 2017). However, less than 1 percent of patients have autosomal dominant inherited Alzheimer’s disease (DIAD) which has a much earlier age of onset, around 45 years old. Genetic mutations in the genes encoding APP, PS1, and PS2 that cause overproduction or formation of an aberrant form of Aβ are found in this group (Levy et al., 1990; Masters et al., 2015).

At the cellular level, AD can be characterized by the appearance of extracellular plaques from accumulations of insoluble amyloid beta (Aβ) filaments, intracellular neurofibrillary tangles of hyperphosphorylated tau and neuroinflammation (Selkoe, 2012). It appears that AD is not only one or two types of diseases, but is rather a group of diseases with similar APP and Tau pathologies that are triggered by different mechanisms (Sery et al., 2013). Among them, the most popular theory is the amyloid cascade hypothesis and detection of the accumulation of Aβ is now available in MCI and AD patients using cerebrospinal fluid biomarkers and PET. However, it is still difficult to find stable biomarkers which could help to discover this disease much earlier, and the exact mechanisms of AD are still unknown. Now the number of studies examining epigenetic mechanisms playing in the etiology of AD has risen dramatically. With this increased focus, the epigenetic modification in AD has become a highly popular topic. This review summarizes the literature on the role of epigenetic mechanisms in the development of AD and the possibilities for treating AD by epigenetically based interventions.

## Epigenetics Factors in AD

**FIGURE 1:**
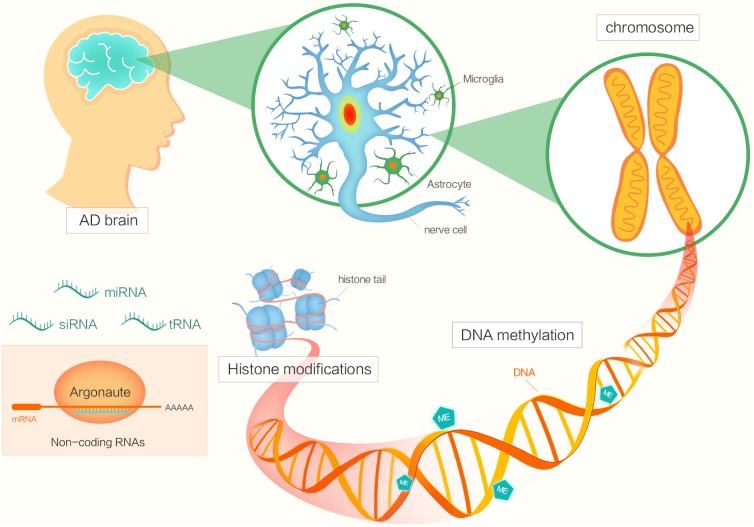
The diagram shows different factors of epigenetics in Alzheimer’s disease.

Epigenetic factors include DNA methylation, histone modifications, chromatin remodeling, and regulation by non-coding RNA (Figure 1) (Morange, 2002; Holliday, 2006; Santana et al., 2017). The functions of epigenetics have been studied in various areas of biology, cancer biology and so on (Jones and Laird, 1999; Champagne, 2013). Now, many researchers are focusing on investigating the potential roles of epigenetics in AD pathogenesis.

## DNA Methylation

DNA methylation modifies cytosine residues by adding methyl groups in cytosine/guanine-rich regions such as CpG islands (Mehler, 2008). The process is initiated by DNA methyltransferases (DNMT), including DNMT1, DNMT2, DNMT3a, and DNMT3b (Kemme et al., 2017). In this section we discuss the possibility that AD could be caused by aberrations in DNA methylation in some certain genes and the potential role of methylation in being a biomarker in AD.

For example, it was found that some cytosines, particularly those at -207 to approximately -182, in the promoter region of the APP gene are mostly methylated and that their demethylation with age may led to Aβ deposition in the aged brain (Tohgi et al., 1999a,b). Methylation of the microtubule-related protein tau (MAPT) gene could also suppress the MAPT expression, which could affect the levels of tau protein (Zhang et al., 2016). Mano et al. (2017) used postmortem brain samples from AD patients and found that the expression of BRCA1 was significantly upregulated, consisting of its hypomethylation. Additionally, BRCA1 protein levels were increased in response to Aβ and became mislocalized to the cytoplasm, in both *in vitro* cellular and *in vivo* mouse models (Mano et al., 2017). Recently, it was found that reduced DNA methylation at the triggering receptor expressed on myeloid cells 2 (TREM2) gene intron 1 caused higher TREM2 mRNA expression in the leukocytes of AD subjects than in the controls (Ozaki et al., 2017). Furthermore, DNA methylation in some other genes also plays a role in the mechanisms of AD and might be a potential biomarker. A study found that DNA methylation (CpG5) of the BDNF gene promoter and a tag SNP (rs6265) have a significant role in the etiology of amnestic mild cognitive impairment (aMCI) and its progression to AD (Xie et al., 2017a). In addition, a 5-year longitudinal study using multivariate Cox regression analysis revealed that elevated methylation of CpG5 of BDNF promoters was a significant independent predictor of AD conversion. This suggests that increased levels of peripheral BDNF promoter methylation may be an epigenetic biomarker indicating the transformation of aMCI to AD (Xie et al., 2017b). Kobayashi et al. (2016) examined the DNA methylation levels of the COASY and SPINT gene promoter regions and found that DNA methylation in the two regions was significantly increased in AD and aMCI as compared to controls. Di Francesco et al. (2015) found that global DNA methylation in the peripheral blood mononuclear cells of LOAD patients was higher compared to healthy controls, and higher DNA methylation levels were associated with the presence of APOE 𝜀4 allele (*p* = 0.0043) and APOE 𝜀3 carriers (*p* = 0.05) in the global population. This indicated global DNA methylation in peripheral samples is a useful marker for screening individuals at risk of developing AD (Di Francesco et al., 2015). Moreover, DNA methylation levels in the NCAPH2/LMF2 promoter region were found to be a useful biomarker for the diagnosis of AD and aMCI (Shinagawa et al., 2016). One study has found that APOE CGI holds lower DNA methylation levels in AD compared to control in the frontal lobe, mostly in the non-neuronal cells of the AD brain (Tulloch et al., 2018). Additionally, PICALM gene methylation was found to be associated with cognitive decline in blood cells of AD patients (Mercorio et al., 2018). Recently, it was discovered that elevated DNA methylation across a 48-kb region spanning the HOXA gene cluster is associated with AD neuropathology (Smith et al., 2018). Moreover, a differentially methylated region was identified within the alternative promoter of the PLD3 gene showing higher DNA methylation levels in the AD hippocampus compared to controls (Blanco-Luquin et al., 2018). All of these indicated that the methylation levels of some genes could be potential biomarkers in AD.

In some postmortem brains or neuronal cells of AD individuals, a lot of work has been done to find more specific evidence. In cortical neurons of a postmortem AD brain, immunoreactivity for 5-methylcytosine (5-mC) was decreased compared to the control (Mastroeni et al., 2010). Levels of 5mC were reduced in the hippocampus, entorhinal cortex and cerebellum of patients with AD (Chouliaras et al., 2013; Condliffe et al., 2014). In hippocampal tissue, Mastroeni et al. (2010) found weak staining for antibodies against DNA methylation maintenance factors in AD cases, contrasting with normal brains. More compellingly, in a comparison of cortical neurons of monozygotic twins, one of whom had AD and the other not, they found extensive co-localization of 5 mC, with three cell-specific markers in the non-AD twin yet an absence of co-localization in the AD twin (Mastroeni et al., 2009). Furthermore, a study identified that numerous AD-related genes, such as MCF2L, ANK1, MAP2, LRRC8B, STK32C, and S100B, have cell-type-specific methylation signatures and document differential methylation dynamics through DNA methylation analysis on purified neurons and glia cells (Gasparoni et al., 2018). Differences were found in DNA methylation in differentiated human neurons with and without Aβ treatment, and that Aβ could change the DNA methylation status of some cell-fate genes (Taher et al., 2014).

## Chromatin Remodeling and Histone Modifications

Chromatin is an assemblage of genomic DNA, histone proteins and associated factors. Chromatin can be dynamically altered by various modifications of histones. Other possible modifications include nucleosome repositioning, chromatin remodeling, and nuclear compartmentalization (Qureshi and Mehler, 2010). Histones are basic proteins that form the building blocks of nucleosomes. Their tails are susceptible to various modifications, including lysine (K), arginine (R), and histidine (H) methylation (Lardenoije et al., 2015). These alterations together constitute the so-called histone code. The histone code is very complex and includes several types of covalent modifications, such as acetylation, methylation, phosphorylation, and ubiquitination, of at least 20 possible sites within the histone proteins (Jenuwein and Allis, 2001).

Among all the above modifications, acetylation is certainly the best characterized post-translational modification of core histones. Histone acetylation is catalyzed by histone acetyltransferase (HAT) while deacetylation is influenced by histone deacetylase (HDAC). It is known that acetylation of histones is associated with enhanced rates of gene transcription, while deacetylation represses gene expression by condensing the chromatin. Here, we want to discuss the role played by HDAC in the AD mechanisms.

Class I HDACs, such as HDAC2 and HDAC3, are expressed at much higher levels than the other HDACs in the memory-associated regions of the brain (Datta et al., 2018). A recent study used a mouse model of AD, which deleted the HDAC1 and HDAC2 genes in microglia cells, leading to a decrease in amyloid load and improved cognitive impairment by enhancing microglial amyloid phagocytosis (Broide et al., 2007). In the CK-p25 AD mouse model, Graff et al. (2012) found that elevated HDAC2 levels epigenetically block the expression of neuroplasticity genes during neurodegeneration, and HDAC2 reduces the histone acetylation of genes important for learning and memory. In another AD mouse model, HDAC2 was found to be strongly expressed in the hippocampus and prefrontal cortex. Neuron-specific overexpression of HDAC2 led to a decrease of dendritic spine density, synapse number, synaptic plasticity and memory formation (Levenson et al., 2004; Guan et al., 2009). In conditions of stress and injury, the level of HDAC2 increases, and this causes decreased expression of genes related to memory and cognition (Levenson et al., 2004; Graff et al., 2012). HDAC3 also plays a vital role in regulating long-term memory formation. Deletion of HDAC3 in the dorsal hippocampus leads to enhanced long-term memory for object location (Mcquown et al., 2011).

In addition to class I HDACs, there is some evidence that class II HDACs are involved to some degree in AD. Class II HDACs are subdivided into class IIa and class IIb (Haggarty and Tsai, 2011). HDAC6, a class IIb HDAC, is elevated by 52% and 91% in the cortex and hippocampus of patients with AD. HDAC6 co-localizes with tau protein in the AD hippocampus, and tau phosphorylation can be decreased by reducing HDAC6 levels (Ding et al., 2008). Govindarajan et al. (2013) using a mouse model of AD, found that decreasing HDAC6 levels could improve cognitive impairment. HDAC4, a member of class IIa, is reported to be associated with synaptic plasticity and memory formation, and loss of it is detrimental to learning and memory (Kim et al., 2012). D’Addario et al. (2017) found that in the peripheral blood cells of monozygotic twins discordant for AD, higher levels of gene expression of HDAC2 and HDAC9 were found in the AD twin. Recently, it was reported that HDAC2 dysregulation could contribute to cholinergic nucleus basalis of Meynert (nbM) neuronal dysfunction, NFT pathology, and cognitive decline during clinical progression of AD (Mahady et al., 2018).

The class III HDACs are called sirtuins, SIRT1-7 (Gray and Ekstrom, 2001). The level of SIRT1 is reduced in the AD cortex, and SIRT1 levels are negatively correlated with the accumulation of paired helical tau filaments (Julien et al., 2009). Tau acetylation can promote pathological tau aggregation, while SIRT1 causes tau deacetylation. There is extensive evidence that SIRT1 reduction is involved in AD tau pathology, and tau acetylation of lysine 28 inhibits tau function via impaired tau-microtubule interactions, promoting tau aggregation (Cohen et al., 2011). Moreover, a significant positive correlation between SIRT1 levels and dementia was found whereby dementia risk increases by a factor of 1.16 due to an increase in the SIRT1 level and a factor of 24.23 due to a decrease in the TLR4 level (Kilic et al., 2018).

Histone acetyltransferases are also involved in the regulation of AD neurogenesis. For example, Tip60, a type of histone acetyltransferase, epigenetically regulates genes enriched in neurons, and its function in axonal transport is mediated by APP. More importantly, Tip60 plays a neuroprotective role in APP-induced axonal transport and functional locomotion defects (Johnson et al., 2013).

In conclusion, a range of studies indicates that histone modifications play a vital role in the development of AD. HDACs can both promote and impair memory formation and cognition.

## Non-Coding RNA Regulation

Non-coding RNAs (ncRNAs) can cause changes in gene expression and the production of proteins and they are involved in the pathology of many neurodegenerative diseases. A recent study used microarray analysis to characterize the expression patterns of circular RNAs (circRNAs), miRNAs and mRNAs in hippocampal tissue from an Aβ1-42-induced AD rat model, and found that a total of 555 circRNAs, 183 miRNAs, and 319 mRNAs were significantly dysregulated (change ≥2-fold and *p*-value < 0.05) in the hippocampus (Wang et al., 2018). In this section, we discuss three aspects of the relationship between ncRNAs and AD.

## miRNAs in AD

In AD, miRNAs are expressed differently in the postmortem brain, blood monocytes and cerebrospinal fluid (CSF). The expression of miRNAs in the hippocampus, medial frontal gyrus and CSF is regionally and stage-specifically altered in AD patient brains using a sensitive qRT-PCR platform, and miRNAs are involved in pathways related to amyloid processing, neurogenesis, insulin resistance, and innate immunity in AD pathogenesis (Cogswell et al., 2008). Levels of members of the miR-29 family, including miR-29a, miR-29b, and miR-29c, are decreased in the brains of patients with AD, and this is related to high BACE1 protein expression (Hebert et al., 2008). It has been reported that miRNA-7, miRNA-9-1, miRNA-23a/miRNA-27a, miRNA-34a, miRNA-125b-1, miRNA-146a, and miRNA-155 are significantly increased in abundance in AD-affected superior temporal lobe neocortexes (Brodmann area 22). These seven upregulated miRNAs are involved in the coordination of amyloid production and clearance (Pogue and Lukiw, 2018). Higaki et al. (2018) suggested that miR-200b/c reduces Aβ secretion and Aβ-induced cognitive impairment by promoting insulin signaling. Moreover, exosome miR-135a and miR-384 were found to be upregulated, while miR-193b was downregulated, in the serum of AD patients compared to that of normal controls (Yang et al., 2018). A study using Tg2576 AD transgenic mice and human AD brain samples found that miR-206 could regulate brain-derived neurotrophic factor (BDNF), which regulates synaptic plasticity and memory (Lee et al., 2012). Moreover, of 8098 individually measured miRNAs in blood cells, six of these miRNAs such as miR-107, miR-125b, miR-146a, miR-181c, miR-29b, and miR-342 were found to be significantly downregulated in individuals with AD compared to controls (Fransquet and Ryan, 2018). These findings indicate that miRNAs are potential biomarkers for AD, and that miRNAs might be involved in the pathophysiology of AD.

## Long Non-Coding RNAs in AD

Long non-coding RNAs (lncRNA) also participate in the pathology of AD. Using a microarray analysis, it was found that in the hippocampal tissue of a rat model of AD, a total of 315 lncRNAs and 311 mRNAs were significantly dysregulated (≥2-fold, *p* < 0.05) (Yang et al., 2017). A type of ncRNA called 51A has been found to regulate the SORL1 gene, which encodes a sorting receptor for the APP holoprotein (Rogaeva et al., 2007). Another study concluded that 51A might increase AD susceptibility by increasing APP formation (Ciarlo et al., 2013). Other lncRNAs are also related to AD mechanisms. For example, RNA 17A was found to be increased in AD. This lncRNA regulates GABAB receptor alternative splicing and signaling in response to inflammatory stimuli (Massone et al., 2011). NDM29 RNA upregulation is accompanied by altered APP modulation and can promote the cleavage activities of BACE (Massone et al., 2012). BC200 RNA is reported to be highly increased in the AD brain in parallel with disease progression (Wu et al., 2013). BC1, another type of lncRNA, is found to induce APP mRNA translation via association with fragile X syndrome protein (FMRP) in mouse model AD brains (Zhang T. et al., 2017). It was recently reported that EBF3 knockdown can reverse Aβ25-35-induced apoptosis in SH-SY5Y cells, and that lncRNA EBF3-AS promotes neuron apoptosis in AD through regulation of EBF3 expression (Gu et al., 2018). Furthermore, the overexpression of Aβ and Aβ-42 in AD could increase BACE1 antisense transcript lncRNA levels, leading to amyloid protein aggregation (Faghihi et al., 2008).

## Other ncRNAs in AD

In addition to miRNA and lncRNA, there are other classes of ncRNAs that play roles in AD that are not as well understood. Recently, it was reported that circRNA-associated-ceRNA networks in an AD mouse model are involved in Aβ clearance and myelin function (Zhang S. et al., 2017). For example, circRNA CIRS-7 significantly decreases in brains with AD. Its effect is to reduce the protein levels of APP and BACE1 (Lukiw, 2013). Y RNA is reported to play an essential pathological role in the altered mRNA landscape of AD (Champagne et al., 2001). Another interesting ncRNA is small NRSE dsRNA, which is a 20bp double-stranded RNA. NRSE dsRNA has been found to bind to the REST complex and activate it. The REST complex protects neurons from oxidative stress and amyloid β-protein toxicity (Rougeulle et al., 1998). The U1 snRNP is reported to show very high expression in neurofibrillary tangles in sporadic AD and early-onset AD (Hales et al., 2014a,b). All these findings point to ways that ncRNAs may take part in the development of AD.

## Applications of Epigenetics to AD Therapeutics

### DNA Methylation-Based Therapy

It has been shown that hypomethylation of AD risk genes such as APP, PSEN1, and PSEN2 has effects on learning and memory. Studies have shown that increases in methyl donor S-adenosyl-L-methionine (SAM) can decrease APP and PSEN1 expression by promoter hypermethylation (Scarpa et al., 2003; Fuso et al., 2005). It may also be relevant that the impaired learning ability induced by lead can be improved significantly by SAM, perhaps by ameliorating the impairments in the long-term potentiation of excitatory postsynaptic potentials that are caused by lead (Cao et al., 2008). Moreover, increasing levels of B12, folate and other methionine sources in the diet can promote methionine bioavailability and reverse elevated expression of APP and PSEN1 (Chan and Shea, 2007; Fuso et al., 2008).

Several studies have focused on finding DNMT inhibitors that could modulate the methylation of AD risk genes. DNMT inhibitors such as azacitidine and decitabine have been approved by the FDA for used in tumors and leukemia (Momparler et al., 1997; Issa et al., 2004). This approach has also been used in some other neurodegenerative diseases, including Friedreich’s ataxia and fragile X syndrome (Chiurazzi et al., 1998; Sandi et al., 2013).

It has also been found that DNA methylation is related to the state of NSC differentiation. As a result, the use of the DNA methylation inhibitor 5-aza-cytidine to treat NSCs could alter DNA methylation, thereby disrupting migration and differentiation (Singh et al., 2009). Loss of Dnmt3a, a type of DNA methyltransferase, was found to impair hematopoietic stem cell differentiation (Challen et al., 2011). Another study showed that intrinsic epigenetic mechanisms play critical roles in the regulation of adult NSC functions. Using methylated CpG binding protein 1 (Mbd1)-deficient mice, it was found that both acute knockdown of Mbd1 or overexpression of fibroblast growth factor 2 (Fgf-2) in adult neural stem and progenitor cells inhibited neuronal differentiation (Li et al., 2008).

### Histone Modification-Based Therapy

Histone deacetylase inhibitors, including valproic acid (VPA), sodium 4-phenylbutyrate (4-PBA), vorinostat (SAHA), trichostatin A (TSA) and nicotinamide, have been shown to produce good results in AD mouse models. We discuss these five inhibitors separately.

Valproic acid has been shown to inhibit Aβproduction in a transgenic AD mouse model (Su et al., 2004). In neuroblastoma and glioma cell lines, VPA could prevent amyloid-β aggregation by increasing clusterin expression (Nuutinen et al., 2010). VPA was also found to decrease Aβ production in transgenic mice, improving their behavioral deficits (Qing et al., 2008). In an AD mouse model it relieved contextual memory deficits via histone H4 acetylation (Kilgore et al., 2010).

4-PBA is reported to decrease β-amyloid production and reverse spatial learning and memory deficits. In the Tg2576 AD mouse model, administration of 4-PBA led to a decrease in tau phosphorylation by increasing GSK-3β (Ricobaraza et al., 2009) as well as increasing intraneuronal Aβ clearance and restoration of dendritic spine densities in hippocampal CA1 pyramidal neurons (Ricobaraza et al., 2012).

SAHA is found to increase the H4K12 acetylation level, thus restoring learning ability in a mouse model (Peleg et al., 2010). In an astrocyte cell line, it induced the expression of clusterin, which might prevent the progress of AD pathogenesis (Nuutinen et al., 2010). In the APPswe/PS1dE9 AD mouse model, SAHA was reported to restore contextual memory by inhibiting HDAC6 in the early stage of AD (Kilgore et al., 2010).

Trichostatin A is found to induce brain-derived neurotrophic factor (BDNF) exon I–IX mRNA in Neuro-2a cells (Ishimaru et al., 2010). Another study of hippocampal neurons also suggested that TSA treatment could be useful at promoter 1 by acetylating histones (H3AcK9/K14) (Tian et al., 2010).

Nicotinamide was found to restore cognitive deficits. Nicotinamide can also increase the acetylation of alpha-tubulin and MAP2c, thereby increasing microtubule stability. These observations suggest a possibility that nicotinamide could be useful in treating AD (Green et al., 2008).

### ncRNA Regulation-Based Therapy

Non-coding RNAs are widely expressed in the brain and show a range of differences between AD and healthy controls. Numerous studies have focused on finding potential treatments using ncRNAs. Studies have found that overexpression of miR-124 and miR-195 could down-regulate Aβ levels by controlling BACE1 gene expression (Fang et al., 2012; Zhu et al., 2012). It is reported that DNA antisense oligonucleotides can hasten the degradation of UBE3A-ATS, which might be an avenue of treatment for AD (Meng et al., 2015). In Tg-19959 mice, knockdown of BACE1 or BACE1-AS transcripts causes reductions in BACE1 protein and insoluble Aβ (Modarresi et al., 2011). In an *in vitro* preparation, MiR-16 was found to be an effective inhibitor of amyloid precursor protein (APP) and BACE1 expression, Aβ peptide production, and tau phosphorylation (Parsi et al., 2015).

miRNA regulators have also been evaluated for use in treating AD. For example, a type of neutralizing inhibitor of miR-206 called AM206 was injected into the cerebral ventricles of an AD mouse model and found to improve memory function (Lee et al., 2012) as well as improving hippocampal neurogenesis and synaptic density. It was reported that RNA interference-mediated knockdown of the long–form phosphodiesterase–4D (PDE4D) enzyme could reverse memory deficits caused by accumulation of amyloid-beta (42) (Zhang et al., 2014). Another study found that miR-34c levels were increased in the hippocampus of AD patients, leading to a suggestion that it might be a biomarker for AD. In an AD mouse model, targeting miR-34 was found to lead to the recovery of learning ability (Zovoilis et al., 2011).

## Discussion and Future Perspectives

Alzheimer’s disease is a progressive neurodegenerative disease which is already a substantial burden on the world. The mechanisms of AD are complex and have been the subject of many theories. We have gained insight into the role of epigenetic modulation, which comprises DNA methylation, histone modification and the effects of ncRNAs. While many differences in these three kinds of epigenetic regulation have been observed between AD patients or models and controls, much work will still be needed to understand this topic and to find potential targets for the treatment of AD.

One of the main challenges for epigenetic therapy is to find molecules that can pass through the blood-brain barrier (BBB). In general, only molecules less than 500 daltons, with fewer than 8 pairs of hydrogen bonds, can move through the BBB. There is a variety of ways of working around this limitation, including BBB disruption, intracerebral implantation and intracerebroventricular infusion (Pardridge, 2005). As discussed above, DNMT inhibitors and HDAC inhibitors are potential therapeutic drugs, and these two kinds of molecules are able to cross the BBB (Hockly et al., 2003; Rai et al., 2008; Kwa et al., 2011). However, several additional factors need to be considered, including efficacy, toxicity, delivery and patient factors. Even though DNMT inhibitors are widely used in treating tumors and leukemia, they are not yet suitable for continuous treatment of AD. Because of their genotoxicity and low stability, they remain at the preclinical stage of development (Cuadrado-Tejedor et al., 2013; Erdmann et al., 2015). However, the clinical side effects of HDAC inhibitors are well known (Subramanian et al., 2010). As an example, the HDAC inhibitor SAHA can induce reactive oxygen species and DNA damage in acute myeloid leukemia cells (Petruccelli et al., 2011). Therefore, more detailed studies are needed to develop safe and effective treatments using DNMT inhibitors and HDAC inhibitors.

Other approaches to ncRNA-based therapy has been examined in recent years. A study showed that a short peptide derived from rabies virus glycoprotein (RVG) assists the transvascular delivery of small interfering RNA (siRNA) to the brain, leading to specific gene silencing inside the brain. Intravenous treatment with RVG-9R-bound antiviral siRNA afforded robust protection against fatal viral encephalitis in mice (Kumar et al., 2007). Other studies have shown that nanoparticle carriers may be a useful method of moving drugs through the BBB (Kreuter, 2001). All of these are methods that may be useful for implementing small interfering RNA therapy.

## Conclusion

In conclusion, we have discussed various epigenetic approaches to the treatment of AD, and new methods have emerged to aid in validating epigenetic targets. However, careful characterization of chemical probes is essential to ensure accurate biological conclusions. A new generation of selective chemical probes needs to be developed to reduce side effects. Studies on epigenetics may lead researchers to new methods of AD diagnosis and therapy. Even though the transition from bench to bedside may be challenging in the near future, current research strongly suggests that epigenetic regulation will become an important tool in AD treatment.

## Author Contributions

XL contributed to the central idea and wrote the initial draft of the manuscript. BJ and LS contributed to refining the ideas, carrying out additional modifications, and finalizing the manuscript. All authors contributed to writing and revising the manuscript.

## Conflict of Interest Statement

The authors declare that the research was conducted in the absence of any commercial or financial relationships that could be construed as a potential conflict of interest.
